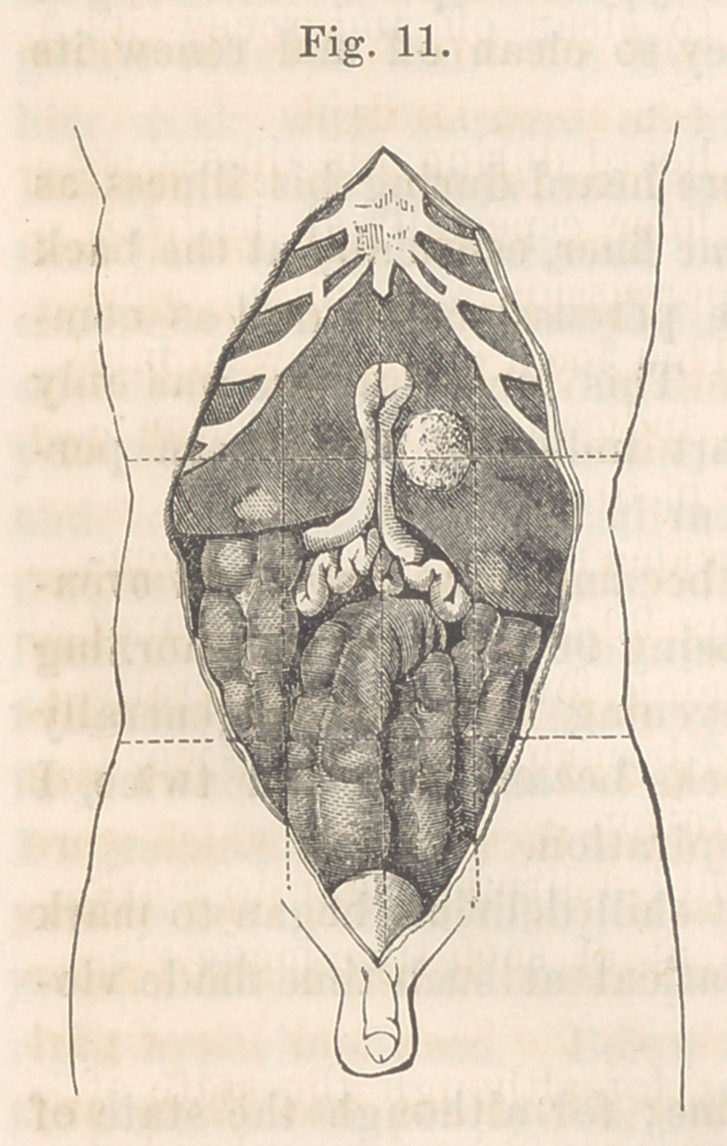# Proceedings of the Pathological Society of Philadelphia

**Published:** 1860-03

**Authors:** 


ART. III.—*Proceedings of the Pathological Society of Philadelphia.*

WEDNESDAY EVENING, NOV. 23d, 1859.

The President in the Chair.

DR. PACKARD stated that he regarded the mammary tumor exhibited at the last meeting as in great measure made up of a structure similar to that of the healthy gland, with an unusually large quantity of fibrous tissue and free nuclei. The thickened and hardened portion of skin close to the nipple, examined in a section, presented a fibrillated appearance, with here and there what seemed to be coils of very fine fibres. In this mass there were also free nuclei, cells of various size, containing one or more nuclei also varying in size, and a few mother cells. Dr. Packard thought, therefore, that to the hypertrophied mammary gland there was superadded, as it were, a cancerous deposit.

DR. WOODWARD said that the portions of the tumor examined microscopically by him were small slices cut from various parts of the diseased mammary gland, and a slice from the greatly thickened and indurated skin near the nipple. In these he found the following conditions:—

*Gland.*—The whole stroma of the gland was the seat of an abundant new formation of connective tissue, as indicated by the presence of numerous spindle-shaped, embryonic elements. For the most part these elements were quite symmetrical and isomorphous with those of ordinary innocent connective tissue formations. When the cut surface of the gland was scraped, no creamy characteristic "juice" exuded, but the slightly turbid and richly albuminous liquid obtained presented the above elements with innumerable clear, transparent, free nuclei, and some extremely delicate dimly-granular nucleated cells.

The *nuclei*, many of which sufficiently resembled those of the healthy gland tissue, varied in diameter from $\frac{1}{200}$ to $\frac{1}{100}$ of a millimeter, or were even a little larger. Their nucleoli were sometimes dot-like, sometimes vesicular.

The *cells* inclosed similar nuclei, and were from one and a half to two or even three times the size of the nuclei.

When thin sections were carefully cut and examined it was found that though the larger milk-ducts were quite recognizable in many parts, the characteristic appearance of the gland tubules was much modified by the encroaching of the overgrown stroma. In no way could it be determined in any of the pieces examined that the lobules of the gland were hypertrophied; on the contrary, in many parts of these pieces they were almost wholly suppressed. In such sections irregular depots of small Aschersonian vesicles in great numbers could frequently be seen in the interstices of the white fibrous bundles of the stroma. These depots were not found in all parts of any slice, but a slice half an inch square would always exhibit one or more patches characterized by the presence of many hundreds of such depots. This peculiar condition of its stroma is highly characteristic of the cancerous breast, and taken in connection with the history of the case, the external appearances of the morbid mass, and its anatomy thus far set forth, would compel the classification of this breast among the cancers.

But an examination of the *skin* which it will be remembered was continuous with the breast near the nipple, elsewhere separated from it by some normal adipose tissue, showed most significant conditions. The areolæ between the fibres of the corion were packed with nuclei and nucleated cells. The diseased skin was from one-fourth to one-half an inch or more thick. On scraping it a juice more turbid than that from the gland issued forth. In this were found, besides a few spindle-shaped elements, abundant huge *free nuclei*, $\frac{1}{100}$ to $\frac{2}{100}$ of a millimeter in diameter, containing large nucleoli, together with uni and bi-nucleated granular cells $\frac{1}{50}$ to $\frac{1}{25}$ of a millimeter in long diameter. In the diseased skin, therefore, as well as in the breast, a new formation of connective tissue was progressing But in the skin the unsymmetrical and distorted characters of the individual elements which stamp a connective tissue growth as cancer were well pronounced; not so in the breast, the whole minute anatomy of which nevertheless fully accords with the ideal of a carcinomatous affection. The difficulty of diagnosis in such cases as this arises out of the *slow development* of the individual elements as compared with the rapid and bulky growth of the whole morbid mass, in consequence of which the characters called cancerous are not profusely manifested especially in the gland tissue. And it is worthy of note that it is in cases more or less allied in minute anatomy to this, that microscopic diagnosis has most frequently failed in the hands of the heterologists.

He was therefore decidedly of the opinion that this growth was a cancer, and with all modesty, in view of the present imperfect state of our knowledge of the relation between the special anatomical characters of cancerous growth and the history of individual cases, would venture to express the conjecture that it will prove rapidly recurring and most malignant; an opinion based upon the past history of similar structural conditions observed in other cases.

DR. KELLER coincided with the views expressed by Dr. Packard regarding the nature of the tumor. He entered at some length into the question of the value of a specific cell as diagnostic of malignant growths, and alluded to the recent views of Rokitansky respecting the stroma of cancerous affections and their mode of growth.

*Cancer of the Mesentery and of the Duodenum.*—DR. HARLAN said:—

The specimens on the table are from a German, aged thirty-two, who died in the Pennsylvania Hospital a week ago.

Unfortunately, his history previously to the date of admission is very unsatisfactory. He spoke but little English, and all that could be learned from him was that he had been employed in a sugar factory and continued steadily at his work, and in good health, up to six or eight months ago, when he began to fail in strength and flesh, and first noticed a tumor in his abdomen. It gave him but little pain until within about six weeks before he applied for admission to the hospital. Since this time he has always had pain across the abdomen varying in intensity.

At the time of his admission, on the thirty-first of October, he was much emaciated, had an anxious, haggard expression of countenance, and his skin and conjunctiva were of a deep-yellow color. His bowels were rather costive, the evacuations soft and very dark. The urine was natural in quantity, but deeply tinged with bile. In the upper part of the umbilical region, and extending into the epigastric, was a tumor apparently the size of an infant's head. It was very evident on superficial inspection, unyielding to the touch, and slightly movable. There was not much tenderness on pressure, and no tympanites, dropsy, or vomiting. The treatment was necessarily palliative, and consisted of the administration of opiates, tonics, and stimulants, and of occasional purges and injections; the latter gave most relief, and he used frequently to beg for their repetition. He became rapidly weaker and more emaciated. Toward the close of his life the pain was more intense, and the tenderness much greater, and two days before his death vomiting and slight tympanites were added to his symptoms.

A *post-mortem examination* was made fifteen hours after death. In the chest there were very firm pleuritic adhesions, but no signs of recent inflammation. The lungs were normal, with the exception of a deposition, in the lower part of the right, of the size of a small marble, and of nearly the consistence of cartilage. The abdominal viscera were almost glued into one mass by adhesions to each other and to a large tumor of the mesentery. There was a small amount of purulent fluid in the abdominal cavity. The liver, right kidney, pancreas, and spleen were apparently healthy. At the pyloric orifice of the stomach, on its inner surface, there was a tumor about four inches in diameter and an inch in its greatest thickness, with its surface depressed and its edges raised and inverted, and with some appearance of ulceration. The duodenum was firmly adherent to the liver, and a few inches from the stomach passed directly through the mesenteric tumor from side to side. This tumor, which is about six inches in its transverse diameter and four in its antero-posterior, and irregular in surface when first taken from the body, had a deep-yellow color, and a consistence firmer than that of a healthy brain. There were a number of other tumors varying from the size of an orange to that of a walnut, some of them entirely surrounding the intestines and having small ulcerations on their inner surfaces. The calibre of the intestine was not perceptibly diminished by the tumors; in one instance it was decidedly increased. The left kidney contained a deposit apparently similar to the matter found in the mesentery. Specimens taken from the deposit in different parts presented the same microscopical appearances; a few spindle-shaped, but principally irregular, round, and oval cells with from one to three or four nuclei.

DR. HARRIS spoke of the extreme difficulty of making a correct diagnosis of abdominal tumors by palpation and percussion, particularly in relation to the primary seat of the disease, and related this case in proof:—

Some thirteen or fourteen years ago, I cannot say exactly when, as I have no written record of the case, I met with an instance of abdominal tumor in an Irishman about fifty years of age. The diseased growth occupied the left upper part of the umbilical region, was very hard to the touch, slightly movable, gave but little increase of pain upon pressure over what was at all times experienced, and handling it did not occasion any increased nausea. The prominence of the tumor was considerable, and the abdominal walls were lifted up above their natural level so as to make a projection as large as the half of an orange.

The prominent symptoms of the case were nausea with occasional vomiting, constant pain in the region of the tumor, entire loss of appetite, insomnia, and a red tongue deprived in spots of large patches of epithelium. Quite a number of eminent physicians saw this patient and made their conjectures as to the organ most affected by the diseased growth. Some supposed the stomach to be the chief site of the abnormal production; others seated the disease in the left extremity of the liver; another thought the spleen was the organ involved; another the pancreas; and another the left kidney. After some months of suffering the man died, and much interest was felt in the post-mortem examination on account of the various opinions which had been expressed with regard to the seat of the tumor. Concerning the nature of the malady but one opinion had been advanced, and that was, that it was cancer.

*Autopsy.*—The stomach was first examined and found healthy; the spleen proved to be normal in every respect; so also the liver, pancreas, and left kidney, which were all removed and dissected before the tumor was touched. The tumor itself was then particularly examined as to its attachments, and it proved to be an enlarged mesenteric gland, of an oval form, slightly flattened laterally, and nearly as large as the egg of an ostrich. It had not given rise to any peritoneal inflammation, neither had it formed any adhesions to the viscera in contact with it. When more minutely inspected it proved to be scirrhus. The brain, heart, and lungs were not examined.

DR. WOODWARD called the attention of the Society to the difficulty in diagnosticating between abscesses in the abdominal walls and visceral tumors. He had had, not long ago, a patient under his care in whom a large swelling was felt in the neighborhood of the spleen, the true nature of which was not discovered until after the occurrence of a chill, and the evident signs of fluctuation in the mass. The tumor was for a long time hard and extremely resistant to the touch. The patient, a young woman, lost strength and flesh, and passed very uncomfortable nights. The idea was at one time entertained that the cause of all the suffering was a malignant tumor of the spleen, but rapid recovery and immediate relief from the more urgent symptoms took place after the discharge of a large quantity of laudable pus. The abscess when carefully probed was not found to communicate with the peritoneum.

DR. GROSS observed that abscesses which originated in the internal organs and opened externally might readily be mistaken for abscesses in the abdominal parietes. In doubtful cases, a careful examination of the matter evacuated was the only sure criterion as to whether or not the abdominal walls alone were implicated. He further thought, that if a surgeon were uncertain as to the nature of an abdominal tumor, it might be proper to pass an exploring needle.

DR. KELLER was of opinion that in cases of abdominal swelling even the exploring needle might be a fallacious guide, and related the following case of *distention of the bladder similating dropsical effusion, in which the bladder was punctured without evil results to the patient*:—

Mrs. B., about thirty-two years of age, and of uncommon fair hair and skin, had been delivered twice of large, healthy children, the youngest of which is now a boy three years of age. Four months ago she had a miscarriage, and was again three months pregnant when I was called to see her, on the twenty-eighth of July, on account of a sense of bearing down pain on the bladder, with an inability to pass water. I found a slight prolapsus vaginæ, and the membrane itself tense, as if some strong pressure were forcing it outward. In introducing the female catheter I had some spasmodic action to overcome, but the urine ran off freely after I had passed the whole length of the instrument. I repeated the same operation every day, and sometimes twice a day, until the first of August, when the patient assured me that she could urinate with ease. On the fourth of the month she complained again of vesical pain, and her digestion was disturbed and she had swelled feet. She showed me half a chamberful of urine which had come from her during the night. On the left side of the pubis a large tumor could be felt; it was round, fluctuating, and of a real cystic nature. This swelling increased daily. On the twelfth of the month I examined the tumor, with a friend, to determine its true character—the tumor had grown to more than the size of a man's head; the upper end reached three inches above the umbilicus. In puncturing with the exploring needle we obtained a fluid coagulating when heated. A day or two afterwards I resorted again to the use of the catheter, notwithstanding the protestations of the patient that she was able to urinate, thinking that an empty bladder would certainly diminish the pressure on the surrounding parts. I introduced every day the catheter its entire length, and always drew off a great deal of water; after each introduction it seemed to me as if the tumor diminished. On the twentieth, I used a long gum-elastic catheter, in part to teach the patient how to use it herself and in part to see how far it would enter the bladder; its whole length entered, and more than double of the usual quantity of urine passed—in fact, nearly two small chambersful. The tumor very quickly disappeared. After this favorable result I introduced the catheter regularly myself every six hours; and for several days there was always a considerable quantity of urine passed after each introduction. By the twenty-seventh of the month the patient had perfectly recovered; she could pass her urine freely at will, and ate and slept well; the œdema of the lower extremities had entirely disappeared.

*Report of Committee on Specimen of Ossification of the Pleura.*—The committee to whom the specimen of bony deposit in the pleura, exhibited by Dr. H. Lenox Hodge, at the last meeting of the Society, was referred for examination, would respectfully report:—

That they found the position of the adventitious mass to correspond with the fissure between the two lobes of the left lung. The pulmonary pleura was adherent to the costal pleura, but could be dissected off; it was perfectly smooth and normal in appearance. The bony mass was enveloped in white fibrous tissue, thicker on its external than on its inner surface, the fibres being as it were radiated. As the costal pleura could not be dissected off from the inner surface of this fibrous envelope, to which, however, it could be traced, the mass seemed to be imbedded between the two layers of the pleura. Upon fracture of the osseous substance an outer compact shell was seen to inclose a cancelated structure, as in the long bones, and microscopical examination revealed the lacunæ and canaliculi of true bone.

JOHN H. PACKARD,

J. DACOSTA,

H. LENOX HODGE.

WEDNESDAY EVENING, December 14th.

Vice-President, DR. EDWARD HARTSHORNE, in the Chair.

DR. DARRACH exhibited a drawing of elastic tissue found in the sputum of a phthisical patient, and spoke of the value of the microscope as an aid to diagnosis in doubtful cases of pulmonary affection. He had been enabled by its use, in the patient from whom he had obtained the sputum, to declare that a destructive disorder of the lungs was going on.

DR. MITCHELL presented specimens of cancer of the liver and lungs, and promised a detailed history of the case at the next meeting.

---

WEDNESDAY EVENING, Dec. 28th, 1859.

The President in the Chair.

*Cancer of the Liver, Lungs, and Spleen, presenting peculiar Difficulties in Diagnosis.*—DR. WEIR MITCHELL read the following:—

T. H., aged fifty-four, stationer and book vender. The patient, a person of spare habit, a hearty eater and of healthy frame—of a family generally healthy, with liability to dropsy late in life, probably from heart disease. His mother died of an affection supposed to be cancer of the face.

In the spring of 1856, I noticed on the palmar face of his hand a small wart, which was rather sore, and was soon accompanied by several others, which became prominent, and cracked, occasionally bleeding. When these tumors became half an inch or more high he consulted in succession several physicians, who pronounced them cancerous. At length, in June, 1859, he fell into the hands of a quack, who applied a plaster which sloughed off nearly two-thirds of the palmar face of the hand and laid bare the tendons. Greatly alarmed at the pain and suffering which ensued, he came to me. I employed cold water dressings only, under which the raw surface healed kindly, the hand being placed on a splint. Before the inner edge of the wound healed, a new formation of a character resembling those which had preceded it appeared on the inside of the hand. The new growth refused to heal, and was aggravated by every kind of dressing applied to it; I finally pared off the small tumor and cauterized its base.

On examination, its structure proved to be chiefly epithelial, with a firm sub-structure of simple fibrous tissue; and I was led to regard it as an epithelial formation, which outwardly and to the unassisted eye it also seemed to be. Its texture resembled that of a soft, cheesy wart, and was readily torn from base to apex; the little finger was so drawn down by the contracting cicatrice which had involved its flexor tendons that it was quite useless, and as the epithelioma seemed disposed to extend, I consulted Dr. Brinton, who finally removed the little finger and half of its metacarpal bone. The wound healed readily.

On various occasions I had examined the patient as to his general health, and was always told that it was excellent, except that he had lost flesh from anxiety, and that he had had a slight cough for several years. Whenever at any time I examined his chest, he exhibited more or less evidence of bronchitis, but the percussion was everywhere even, and not merely clear, but uniformly resonant or tympanitic in tone. At the time of the operation, about November first, Drs. Brinton, Kane, and myself auscultated his heart, and were in some doubt as to the use of ether, because there was heard a faint mitral blowing sound. It was so trifling that it was not regarded as of enough moment to forbid the employment of the anæsthetic, which was therefore given with ordinary freedom. It will be observed that at this time the patient's bowels were regular, his digestion and appetite good, and that, up to the date of the operation, he was free from visceral pain or annoyance, and was neither jaundiced nor sallow. During the healing of the wound he complained of pain in the splenic region. This pain was very sharp, and was treated by Dr. Brinton, who blistered the region in question, and, I believe, altogether relieved him. On November twentieth he sent for me. I found him weak, with increase of his chronic coughing, especially at night. His sputa were yellow and green, like those of old bronchitis, and at no time had he any gelatinous or currant-jelly like expectoration. Upon close questioning, he complained of loss of appetite, and of having had, within two days, some diarrhœa. His tongue was thickly coated and yellow. At this time he was sent to bed and treated with stimulants and tonics, and with nutritive fluid diet. On the twenty-third, he had a rigor at 12 M., and one of slighter nature about 2 P.M. on the twenty-fourth. Each chill was succeeded by fever and sweat. On the twenty-fifth his pulse was 96, his respiration easy and rapid, his tongue more furred and dryer, his bowels costive. As soon as he was placed in bed I examined his belly with care; it was small and not distended. The veins were a little enlarged, and discharged their blood downward as usual. The liver dullness extended about an inch below the ribs in the right hypochondrium. Below the ensiform cartilage there was tympanitic clearness on percussion, and this extended downward into the usual region of clear percussion over the small intestines. To the left of this resonant epigastric space there was marked dullness, which could be traced down from the left curve of the ribs for about five inches, and thence along the border of the ribs toward the left side, where again was clear percussion. Thus, although the liver appeared enlarged, there was a distinct band of clear percussion in the epigastric space, and as there was neither dropsy, jaundice, or want of bile in the stools, it was presumed that the dull percussion on the left must indicate a disease which could not be regarded as being connected with the liver.

As the case advanced, and the patient became emaciated, the left and middle part of the epigastric space exhibited at each inspiration a swelling which corresponded to the dullness on percussion above described. This prominence disappeared during expiration, and followed the motions of the diaphragm. It gave to eye and touch the impression of a circumscribed rounded tumor, lying between the liver and spleen, and having to its right side a clear percussion space, presumed to be due to the stomach, or, at all events, to indicate that to the right of this point lay the boundary of the enlarged liver. On the left side the usual percussion dullness marked the splenic region, below which the percussion was tympanitic.

The progress of the case was, from this time, a downward one. Without headache or local pains, or iliac gurgling, or tympanitis, or eruption, the case was plainly typhoidal, but not a typhoid fever. The bowels were costive throughout, and when moved, presented stools which were dark—or light colored at times—but not of typhoid aspect. The tongue was dry, dark, and coated, with a tendency to clean off and renew its epithelia from time to time.

The bronchial râles above alluded to were heard during his illness as they were in health, except that they became finer, especially at the back and lower half of the left lung, where the percussion was dull as compared with the same region on the right. This local dullness was only a comparative symptom, and had the part indicated alone been percussed, it would have been described as clear in tone.

After the thirteenth day these symptoms became more grave, and evening exacerbations were noticed, the pulse being 90 to 100 at the morning visit, (10 A.M.,) and 120 to 130 in the evening fever, which generally disappeared after 12 at night; both cheeks became red, and twice, I think, the fever was followed by some perspiration.

About the fourteenth day from the first chill delirium began to mark the height of the evening fever, and the patient at such time made violent efforts to leap out of bed.

This delirium was not seen in the daytime; for although the state of his mouth made thick his speech, when the words could be comprehended they were usually apt and correct.

On the fifteenth day the patient was worse; his pulse more rapid and more feeble. Emaciation increased, the tendons twitched, he plucked at the bed-clothes, and lifted himself on his elbows, at the same time staring about him. Meanwhile the face became pinched, cold local sweats broke from him, and, without much increase of tympanitis, without diarrhœa, and without convulsions, he died quietly on the evening of the eighteenth day from the time of the first chill.

But one point remains unnoticed. The urine examined before the fatal illness, and during it, exhibited no albumen at any time, and was never retained, or in any way the cause of annoyance.

On the last day of the illness the patient was seen by Dr. Da Costa, who agreed with me in regarding the case as a typhous condition, arising from some organic malady. We came to the conclusion that the tumor, which we could now feel and could limit by percussion, was in all probability omental. At this very time the chest percussion was emphatically clear, and even tympanitic, and the middle epigastric space was, as before, so distinctly tympanitic as to force us to note this clear spot as the right boundary of the tumor.

*Post-mortem section* by Dr. Kane, about twenty hours after death. Present, Dr. Da Costa and myself. Post-mortem rigor well marked. Body emaciated; no jaundice.

*Abdomen.*—Belly discolored. The locality of the swelling no longer prominent. On dividing the abdominal walls, the viscera were found to be singularly disarranged. The transverse colon was pushed downward and twice doubled in zigzag across the middle of the belly, as represented in the drawing made by Dr. Kane while the parts were undisturbed. The colon, which was dotted with dark-green oval spots, still lay above the small intestines which it had thrust down before it.

Fig. 11.

The cardiac end of the stomach was nearly in place, but the pylorus was pushed down so as to straighten out the small curvature of the stomach and place the larger part of the greater curvature in the left hypochondrium. The left end of the stomach lay therefore in the left hand V of the W formed by the dislocated colon; and had a plumb-line been dropped from the stomachal end of the œsophagus through the stomach, when the patient was erect, it must have fallen on or near the pyloric opening.

From the pylorus the duodenum turned abruptly up and to the right, so that its first portion and a part of the jejunum lay in the epigastric region, within an inch or so of the end of the ensiform cartilage. This loop of intestine reposed in a deep furrow on the front surface of the liver. From this again the duodenum turned downward to end in the small intestines.

The liver, perhaps doubled in weight, descended on the right about one and a half inches below the ribs. In the epigastric region, across which it passed into the left hypochondrium, the organ was deeply indented, and from this notch ran backward a deep furrow in which lay the duodenum as above described; to the left of this furrow the liver descended below the ribs to some distance, and at this part was a large prominent encephaloid tumor about four inches in diameter.

On further inspection, four such masses were found in the liver, thus constituting the rarest of all the forms of hepatic cancer. The tissue of the liver was partially degenerated into fat, but in general the organ was normal. The gall-bladder was full, but not distended.

The spleen was small, and contained several small, soft, cancerous nodules. The pancreas and kidneys were healthy. The left supra-renal capsule had undergone degeneration, and was yellow without, and within full of a thin, brown, grumous matter, but not cancerous. The mesenteric glands near the liver were enlarged and cancerous, but elsewhere they were normal. The peritoneal cavity contained no fluid.

*Thorax.*—This cavity was observed by those present at the section to be singularly resonant on percussion. The heart was small and slightly affected with fatty metamorphosis. The valves closed perfectly; but one of the mitral curtains was irregularly thickened, and the ring from which spring the aortal valves was also a little more stiff and unyielding than usual.

The lungs were large. When removed they floated in water; wherever struck they emitted a clear, rather tympanitic sound. On incision they were found to be equally studded in every part with innumerable nodules of soft cancer of a yellowish-white tint, and varying from the size of a millet-seed to that of a walnut. Between these spots, which were in every half inch of the lung, or even nearer together, the lung tissue seemed healthy. More or less congestion existed between the cancer masses; but although this was more notable at the back and lower part of both lungs, neither in this nor in other situations was there any marked œdema of the pulmonary tissue.

So general was the distribution of the cancer, that it was impossible to say whether or not the lung was emphysematous. If it was so, the dilatations could not have been large. The pleural membrane was healthy, but some of the nodules raised its pulmonary layer into prominences easily seen and felt before the lung tissue was incised. The blood was everywhere fluid and dark.

*Remarks.*—The case which I have reported is one of singular interest, and in some particulars will repay a comparison of symptoms with pathological appearances. The existence of a tumor in the abdomen was not suspected until the last illness. The only pain ever felt was in the left side, remote from the liver, and in despite of the great displacement of the viscera, the appetite and digestion remained good, and the fecal evacuations regular. There was no jaundice, or ascites, or œdema, and but a very slight enlargement of the abdominal cuticular veins. In despite of the utter absence of hepatic signs, or of symptoms of cancer of the liver, its presence might have been suspected after the existence of an abdominal tumor had been once ascertained, if it had not been for one circumstance. Whenever the belly was examined, a space of clear percussion was found in the middle epigastric space, so that it was supposed to mark the true limit of the dullness which existed over the tumor. As we have seen, this clear sound was owing to the duodenum and jejunum, which were drawn or forced up into the fissure formed on the right and left by the enlarged liver and covered in front by the anterior abdominal walls. It was physically necessary that this groove should be filled, and consequently the atmospheric pressure upon the more yielding portions of the belly seems to have driven into the void the intestines above referred to. Hence the clear sound and the error which, with the absence of pain, jaundice, and dropsy, induced us to refer the growth to other organs than the liver.

If the hepatic cancer had been more clearly expressed, it is probable that the general bronchitis might have been attributed to its correct cause. But of this even I am not sure; and I am certain that when a physician sees a general bronchitis with a clear and even percussion throughout both lungs, and with only occasional nocturnal dyspnœa, and when, moreover, a mitral murmur seems to explain this last symptom, under these circumstances, I say, few are likely to arrive at a correct diagnosis. The knowledge of the state of the liver was the only guide.

Dr. Walshe describes pulmonary cancer as thus characterized:—

Pain almost invariably present, and this due, at least such is his inference, to inflammation of the pleura. In my case there was no pleurisy and no pain, except temporarily, and late in the disease.

Dyspnœa is a very constant symptom. In this case it did occur at night, but was not very marked, and did not continuously affect the patient.

Cough was present in this as in all other cases of pulmonary cancer.

Unlike all cases of this kind, hitherto known to me, in this one the sputa was altogether uncharacteristic.

The decumbency in this as in most asthenic cases was dorsal during the last illness. Before this it was observed that the habit of health—sleeping on the right side—prevailed.

At page 348, Dr. Walshe recognizes the disseminated nodular form of cancer of the lung, even when the nodules are very numerous, as commonly giving rise to no symptoms, and as not usually altering the resonance of the chest.

This is also the case in some instances of acute tubercle, and in both diseases it is probably due to an emphysema developed *pari passu* with the constitutional and pulmonary malady.

*Rupture of the Coronary Artery and Hemorrhage into the Pericardium.*—DR. HARLAN, on exhibiting the heart, said:—

A sailor, twenty-two years of age, was admitted into the Pennsylvania Hospital on the second of December. He stated that he had been taken sick six or eight weeks ago at Havana, that no physician had seen him, but that his captain had pronounced the disease to be yellow fever. He never left his berth until the vessel came to port, when he was carried immediately to the hospital.

At the time of his admission he was much debilitated, complained of difficulty of breathing and a feeling of constriction across the chest; he had a frequent, feeble, and very irregular pulse; his bowels were costive, and his appetite poor. His lips were purple, and his skin was cold and damp. The area of dullness on percussion over the heart was much increased; the sounds of the organ were scarcely audible. There was dullness over the back of the chest, and egophony. His face was a little puffy, and there was some anasarca of the lower extremities.

In the course of a few days vomiting occurred, accompanied with epigastric tenderness and almost fatal prostration; it yielded temporarily to remedies, but recurred on several occasions, and each time left the patient more debilitated. The pericardial and pleural effusion continued to increase, and with it the dyspnœa and prostration. He died quite suddenly on the morning of December nineteenth, seventeen days after his admission into the hospital. A few minutes before death he took some wine from the nurse, who did not notice any change in his appearance.

*Autopsy.*—When the thorax was opened, the pericardium appeared to occupy nearly the whole of the left side, entirely concealing the left lung, which was compressed by it. The pleura contained a pint and a half of reddish fluid, but presented no signs of recent inflammation. The pericardium contained twenty ounces of a similar fluid and half a pint of firmly coagulated blood. There was no rupture of any of the cavities, and the great vessels at their origin were sound. The pericardium was much thickened, and both its cardiac and reflected surfaces were nearly covered with an abundant deposit of coagulable lymph. The right side of the heart was attached to the pericardium by fibrous bands. At the base on the left side the marks of inflammation were most decided, the coating of lymph was thickest there, and the muscular tissue was softened; at one point there was a depression, presenting the appearance of a ragged ulceration and containing a piece of a clot. A probe passed gently into the coronary artery came out at this point, and, on cutting down upon it, the artery could be plainly seen to open directly into the pericardium.

So far as I have been able to learn, this case is unique. It is not a very uncommon thing to find fluid blood mingled with the pericardial effusion. In hemorrhagic states of the system there may be a passive hemorrhage from the pericardium, as there occasionally is from other serous surfaces. Lebert says that blood is sometimes found mingled with the hydrops pericardii in cases of general dropsy, especially of Bright's disease; and when inflammation attacks a newly organized false membrane, hemorrhage is said to result from the rupture of its tender vessels. In all these cases the blood is fluid and comparatively inconsiderable in amount, but a quantity of clotted blood must result from some decided organic lesion, and is an accident of much more rare occurrence. Under the head of "Anomalies of the Contents of the Pericardium," Rokitansky mentions "blood in a fluid or coagulated state," and says "that it is almost always an arterial extravasation, and has been deposited by the spontaneous rupture of the left ventricle or by a laceration of the origin of the aorta, occasionally as the termination of an aneurism." Lebert refers it to a rupture of one of the cavities of the heart, or of aneurism of the aorta, and mentions, as a very rare case, the rupture of an aneurism of the coronary artery. This is the only reference I can find to the coronary artery in connection with the presence of blood in the pericardium.

Though ulceration of the substance of the heart is considered an uncommon result of inflammation, it is well known to happen occasionally. Most cases of rupture of the cavities, according to Laennec, are the result of ulceration; and Hasse thinks that rupture of the heart is of a passive nature, and that, in many cases reported as spontaneous rupture, ulceration must have existed, though overlooked. Hope, Laennec, and Andral have all seen abscesses of the heart as the result of partial inflammation of its substance occurring in cases of pericarditis; and Hope says that ulcers are less rare than abscesses, though they generally commence in the endocardium and extend outward. The appearances of the ulceration in this case were not very well marked; the edges were rugged and fused with the loose, shaggy deposits of lymph entangling pieces of clot; and if the progress of the ulcer had not been stopped before it reached the cavity, by death, it would very possibly have been overlooked, and the case reported as one of rupture from softening.

As the diagnosis of yellow fever has no other proof than the dictum of a sea-captain, the probability is that the heart was originally the seat of disease, and that the pathological changes were the result of an inflammation of two months' standing.

*Typhoid Fever; Penetrating Ulcer.*—DR. HARLAN next exhibited a penetrating ulcer of the intestine.

John Boyle, an Irish laborer, aged twenty-two, was admitted into the Pennsylvania Hospital, on the sixth of September last, for typhoid fever. There was nothing very remarkable about his case, except that the convalescence was unusually slow, and that he had several relapses without any assignable cause. He was treated with quinine and stimulants, and three weeks after admission was put upon the use of oil of turpentine, which was continued for about a month. His last relapse occurred in the latter part of October; since then he improved slowly until two weeks before death, when he was able to walk about the wards for a greater part of each day. His appetite was good, his tongue clean and moist, and his bowels were moved with remarkable regularity once a day, but there were always a dullness of intellect and an unnatural expression of countenance, which showed that all was not right; he always seemed like a sick man. On the evening of the sixteenth of December, when I made my visits to the wards, he was sitting as usual in his place, and said he felt pretty well. During the night he was attacked with pain in the abdomen, and the next morning I found him much prostrated, his pulse frequent and feeble, his abdomen slightly tympanitic and tender to the touch, his tongue brownish and perfectly dry, and his face and chest bathed in perspiration. On the morning of the eighteenth he was suddenly seized with vomiting, during which he died.

*Autopsy* ten hours after death. Lungs, heart, and kidneys healthy; liver slightly enlarged; spleen enlarged and softened; membranes of the brain congested—brain itself slightly so, but very firm. Peritoneum presented well-marked signs of recent and extensive inflammation. An accidental cut of the intestines prevented us from determining the existence of extravasation. Numerous ulcerations of Peyer's glands extending for four or five feet in close succession from the cæcum upward, many of them apparently undergoing the process of cicatrization. In one ulcer, nearest the cæcum, there was a small oval perforation with smooth edges.

The point of interest in this case is the unusual length of time intervening between the commencement of the disease and death by perforation. Dr. Gerhard has never seen or heard of perforation after anything like so long a period, and the longest period that I can find, mentioned by Louis, is forty-two days. This man died three months and a half after he came to the hospital, most probably at least four months after the commencement of the disease.

DR. STILLÉ made some remarks with regard to the frequency with which the final perforation of the bowel was caused by imprudence in diet, and mentioned a case in which it had been brought about by the patient's eating a large quantity of ginger-bread, more than a month after the febrile symptoms had passed off.

DR. DA COSTA mentioned that a case had come under his notice, in which perforation had occurred after a lapse of seven months. The perforation followed the eating of a large quantity of chestnuts. The patient, a young man, had had a very severe attack of typhoid fever.

DR. LENOX HODGE exhibited an *Incised Wound of the Intestines*, taken from a patient who was brought to the Pennsylvania Hospital, December twenty-fourth, with a stab in the right inguinal region. About fourteen or sixteen inches of the small intestines, wounded in three places, protruded. These wounds were three-fourths of an inch in length, and partially closed by the everted mucous membrane. They were stitched with the continuous suture of silk, the intestine returned, and the external wound closed by the interrupted suture of lead, carried only through the skin. As soon as the gut was returned, the man, almost dead before, began to react. He lived for forty-two hours. There was a great effusion of fluid blood, at least three pints, in the peritoneal cavity.

*Softening of the Right Side of the Brain; extensive Chronic Hydrocephalus on the Left Side.*—DR. KELLER exhibited, through Dr. Packard, specimens of a cerebral lesion, and of atheromatous deposits in the arteries. Dr. S., fifty-one years of age, a bachelor from Germany, was, at six o'clock in the morning, at the beginning of February, seized with paralysis of the left arm and leg. He was a man of very regular habits, and of fanatical love for anything instructive; a great scholar in botany, geography, and languages, but deficient in those sciences which are not, for the most part, dependent on memory. The paralysis was soon relieved, and he was able, four weeks afterwards, to go out again and to use his arm tolerably well.

About the middle of March, in consequence of a fatiguing walk the previous evening at a late hour, and an attack of diarrhœa during the night, complete paralysis returned. The paralysis of the left arm and leg seemed to get somewhat better during the summer, but still the movements remained imperfect. As the weather got cooler the limbs contracted, and he was entirely unable to move in the least. In December his ideas became confused, and he died delirious, on the 26th of December, 1859, after eleven months' sickness.

*Autopsy* made by Dr. Packard twenty-four hours after death.—Body rigid. The bones of the cranium were thick and nowhere attached to the dura mater. A great deal of serum in the subarachnoid space and ventricles. Vessels of pia mater highly congested. Cerebral substance generally normal. Left ventricle very much enlarged, so as to extend over nearly the whole length of the cerebral hemisphere. Right ventricle of normal size, but in the corpus striatum of that side there was a cavity of the size of a marble, opening into the ventricle, and surrounded by a spot of softening of the cerebral substance about as large as an egg. Under the microscope, this softened brain-matter was found to present the pouching of the tubuli in a more marked degree than the adjacent portions did. It contained also numerous oil-drops, and a number of large, dark, conglomerate-looking corpuscles, such as are usually present in softened brain-substance. The basilar artery was completely blocked up with clots, as was also the right carotid. These vessels were atheromatous, and the basilar artery was aneurismally dilated. From their appearance, these clots seemed to have been formed some time previous to death. The heart was large and pale, and contained a very voluminous soft black clot, and some fluid blood. Under the microscope, it was seen to be very slightly fatty. Atheromatous patches in every stage of formation, and of every size, existed throughout the aorta, and in the iliacs as far as traced; all the arteries given off from the aorta presented the same change. At some of these patches in the aorta the internal coat was eroded. The valves of the heart were healthy, the bicuspid being, however, somewhat thickened. Lungs perfectly healthy, with very slight pleuritic adhesions at their apices. Liver very slightly fatty. Spleen and pancreas normal. Kidneys large and pale, and softer than usual, but without marked change. Supra-renal capsules separated from them by a large quantity of fat, and not satisfactorily examined. Intestines not examined; apparently normal.

## Figures and Tables

**Fig. 11. f1:**